# Prdx6 regulates *in vivo* myeloid cell development via redox control during *Xenopus* embryogenesis

**DOI:** 10.1080/19768354.2025.2533823

**Published:** 2025-07-16

**Authors:** Minjoo Kim, Hyun-Kyung Lee, Hongchan Lee, Hyun-Shik Lee

**Affiliations:** KNU G-LAMP Project Group, KNU Institute of Basic Sciences, School of Biotechnology, BK21 FOUR KNU Creative BioResearch Group, College of Natural Sciences, Kyungpook National University, Daegu, Republic of Korea

**Keywords:** Peroxiredoxin 6, myeloid cell, reactive oxygen species, redox regulation, *Xenopus laevis*

## Abstract

Peroxiredoxin6 (Prdx6) is a bifunctional antioxidant enzyme with both peroxidase and phospholipase A₂ activities. Although its molecular roles are well established, the developmental role of Prdx6 remains poorly understood. To address this gap in the literature, this study aimed to examine the *in vivo* function of Prdx6 in primitive myelopoiesis using *Xenopus laevis* embryos. We found that *prdx6* is specifically expressed in myeloid progenitors originating from the anterior ventral blood island during early embryogenesis. Knockdown of *prdx6* significantly reduced the number of myeloid cells, without affecting their migration ability. Embryos depleted of *prdx6* exhibited elevated levels of reactive oxygen species (ROS) and decreased cellular proliferation. Co-injection of morpholino (MO)-resistant *prdx6* mRNA or treatment with N-acetylcysteine (NAC) successfully restored both ROS levels and myeloid cell numbers, suggesting that Prdx6 supports primitive myeloid cell development by maintaining redox homeostasis. These findings reveal a novel role of Prdx6 in ROS-dependent proliferation of myeloid progenitors during early vertebrate development.

## 1. Introduction

Peroxiredoxins (Prdxs) are a family of thiol-specific antioxidant enzymes that neutralize peroxides through cysteine-mediated redox reactions. Based on the number and function of their catalytic cysteine residues, they are classified as either 1-Cys or 2-Cys Prdxs. Among the 2-Cys Prdxs, Prdx1 to Prdx4 are typical representatives, whereas Prdx5 is regarded as atypical (Barranco-Medina et al. 2009). Prdx6, a 1-Cys enzyme, is evolutionarily conserved and ubiquitously expressed across a diverse range of species (Wood et al. 2003). Despite its structural and functional similarities to other members of the Prdx family, Prdx6 exhibits notable distinctions. It contains a single conserved cysteine residue and uniquely requires glutathione (GSH), rather than thioredoxin, for activation of its peroxidase function (Manevich et al. 2004). During catalysis, Prdx6 forms a heterodimer with GSH. Additionally, it possesses a Ca^2+^-independent phospholipase A_2_ (aiPLA_2_) domain, rendering it bifunctional with both peroxidase and phospholipase activities. In mammals, the aiPLA_2_ activity of Prdx6 plays a key role in repairing oxidative damage to cellular membranes (Chen et al. 2000; Zhou et al. 2016).

Although the molecular and cellular functions of Prdx6 are well documented, its developmental role, particularly during embryogenesis, remains insufficiently explored. To address this knowledge gap, we investigated *prdx6* mRNA expression in *Xenopus laevis* embryos. We observed its specific localization in primitive myeloid cells, which play a crucial role in early immune defense during embryonic development.

Hematopoiesis occurs in two distinct waves. The first, known as primitive hematopoiesis, produces a transient population of early functional blood cells (Ciau-Uitz Walmsley and Patient 2000; Agricola et al. 2016). The second wave, termed definitive hematopoiesis, generates self-renewing hematopoietic stem cells that colonize various hemogenic sites throughout later development and into adulthood (Chen et al. 2009). Although less extensively studied than definitive hematopoiesis, primitive hematopoiesis has been well characterized in *Xenopus* and zebrafish, offering valuable insights into its regulatory mechanisms.

In *Xenopus*, primitive myeloid lineages arise from the anterior ventral blood islands (aVBI), which originate from the dorsal gastrula mesoderm (Kulkeaw and Sugiyama 2012). These embryonic myeloid progenitor cells are evolutionarily analogous to yolk sac-derived myeloid cells in mammals (Chen et al. 2009). During the tailbud stage, they differentiate into neutrophils and macrophages, coinciding with the formation of the vascular network (Sakata and Maéno 2014). Functionally, these primitive myeloid cells contribute to innate immunity by migrating to injury sites and performing phagocytosis, thereby protecting the embryo from infection and injury prior to the onset of definitive hematopoiesis (Costa et al. 2008).

In this study, we investigated the developmental role of Prdx6 in *Xenopus laevis*, a well-established vertebrate model, with a particular focus on myelopoiesis. Knockdown of *prdx6* resulted in reduced proliferation of myeloid cells and elevated levels of reactive oxygen species (ROS), leading to impaired development of primitive myeloid cells. These findings suggest that Prdx6 serves as a critical redox regulator necessary for the proper formation of the primitive myeloid lineage during embryogenesis.

## 2. Materials and methods

### 2.1. *Xenopus laevis* growth conditions and *in vitro* fertilization

All animal procedures were conducted in accordance with the ethical guideffiglines of Kyungpook National University and were approved by the Institutional Animal Care and Use Committee (IACUC) under protocol number (2021-0017). Adult *Xenopus laevis* were obtained from the Korean *Xenopus* Resource Center for Research and maintained at 18°C under a 12-hour light/dark photoperiod in appropriately sized containers. To induce ovulation, female frogs were injected with 1,000 IU of human chorionic gonadotropin (hCG) into the dorsal lymph sac on the evening prior to egg collection. The following morning, females were transferred to 1× high-salt solution for egg collection. Male frogs were anesthetized in 1× benzocaine solution for 5–15 min and then sacrificed for testis extraction. The testes were stored in 1× modified Barth’s solution (MBS) at 4°C. Eggs were washed three times with 0.1× MBS and fertilized *in vitro* using a sperm suspension prepared from the extracted testes. Following fertilization, embryos were de-jellied by swirling in 2% L-cysteine and washed five times with 0.5× MBS. Unfertilized and non-viable embryos were removed under a light stereomicroscope. Healthy embryos were cultured at 15−18°C in 0.5× MBS supplemented with 2% Ficoll 400 (GE Healthcare).

### 2.2. Plasmid construction and mRNA synthesis

Total RNA was extracted from tailbud-stage embryos, and cDNA was synthesized for prdx6 cloning. Primers were designed based on the prdx6 sequence (Accession No. NM_001090847.1) obtained from NCBI and Xenbase. HA-tagged prdx6 cDNA was amplified by polymerase chain reaction (PCR) and subcloned into the pCS107 vector, which was then linearized with AscI (Takara). A morpholino (MO)-resistant mRNA (prdx6*) was synthesized for rescue experiments by introducing seven-point mutations into the wobble positions of codons while preserving the original amino acid sequence, followed by an ATG start codon. HyPer-cyto was subcloned from the pHyPer-cyto plasmid (Evrogen) into the pCS2 + vector and linearized using NotI (Takara). Capped *Prdx6* and *HyPer* mRNA were transcribed *in vitro* using the SP6 mMessage mMachine kit (Invitrogen).

### 2.3. Morpholino oligonucleotides (MO) design and microinjection

A translation-blocking morpholino (MO) targeting *prdx6* was designed and synthesized by Gene Tools. The 25-mer sequence was 5′-TTCGCCTAGCAGGATACCTGGCATG-3′. MOs were injected into the animal hemisphere of one-cell-stage embryos using a calibrated microinjector.

### 2.4. Whole-mount *in situ* hybridization (WISH)

Embryos were fixed at the desired stages in MEMFA solution (4% paraformaldehyde, 0.1 M MOPS (pH 7.4), 1 mM MgSO_4,_ 2 mM EGTA) overnight at 4°C. DNA templates for RNA probe synthesis were linearized with the appropriate restriction enzymes. DIG-labeled antisense RNA probes were transcribed using SP6 or T7 RNA polymerase (Invitrogen). Hybridized probes were detected using an alkaline phosphatase-conjugated anti-digoxigenin antibody (1:1000, Roche) and visualized with NBT/BCIP substrate (Roche). Whole-mount *in situ* hybridization (WISH) was conducted using the following RNA probes: *prdx6* (NM_001090847.1) and *mpo* (NM_001087639).

### 2.5. Reverse transcription – polymerase chain reaction (RT–PCR)

Total RNA was extracted from embryos at developmental stages 1–40 using Isol – RNA lysis reagent (5 Prime GmbH). First-strand cDNA was synthesized using the PrimeScript 1st-strand cDNA synthesis kit (Takara) with the following cycling conditions: 65°C for 5 min, 42°C for 1 h, and 95°C for 5 min.

PCR amplification was performed using gene-specific primers. Products were separated on a 1% agarose gel and visualized using a WiseCapture I-1000 imaging system (Daihan Scientific) (Table 1).

**Table 1.** Primer sequences used for RT-PCR.

Gene	Forward Primer	Reverse Primer
odc	5´–CAGCTAGCTGTGGTGTGG–3´	5´–CAACATGGAAACTCACACC–3´
prdx6	5´–TGGCATCAGGTGTTCGGAT–3´	5´–CTATAAATTATGTCCTTC–3´

### 2.6. Western blot analysis

Embryos were homogenized in lysis buffer (137 mM NaCl, 20 mM Tris-HCl (pH 8.0), 1% Nonidet-P40, 10% glycerol) supplemented with 1 mM phenylmethylsulfonyl fluoride (PMSF), 5 mM sodium orthovanadate, and 1× protease inhibitor cocktail. Samples were heated at 95°C for 5 minutes and subjected to SDS-PAGE on a 12% gel. Proteins were transferred to membranes and probed with an anti-HA-HRP antibody (1:1000, Roche). Signals were detected using an ECL substrate (Cytiva).

### 2.7. *In vivo* imaging of reactive oxygen species (ROS)

To evaluate H_2_O_2_-related ROS levels, 500 pg of *HyPer* mRNA was injected into the animal hemisphere of one-cell stage embryos. The HyPer plasmid used for *in vitro* transcription was obtained as previously described (**Lee et al. 2020**). Embryos were anesthetized with 1:1000 diluted benzocaine and imaged live at the tailbud stage using an Olympus FV1200 confocal microscope. Fluorescence intensity was quantified using ImageJ software (National Institutes of Health; http://imagej.nih.gov).

### 2.8. Phospho-histone 3 (PH3) staining

Stage 22 embryos were fixed in paraformaldehyde and permeabilized in PBS containing Triton X-100. After blocking in 1% BSA and 5% goat serum, embryos were incubated with an anti-phospho-Histone H3 (PH3) antibody (1:2000, Abcam). Following four 1-hour washes in PBS-Triton, embryos were incubated with an anti-rabbit IgG-AP secondary antibody (1:1000, Cell Signaling Technology) and signal was developed using NBT/BCIP (Roche).

### 2.9. Statistical analysis

Fluorescence and staining data from HyPer-GFP and PH3 experiments were analyzed using ImageJ software. Statistical analyses were conducted using GraphPad Prism 9. Data are presented as mean ± standard error (n = 3 biological replicates per condition). Significance was determined using Student’s *t*-test, with thresholds set as follows: *P < 0.05, **P < 0.01, ***P < 0.001, ****P < 0.0001.

## 3. Results

### 3.1. Prdx6 is expressed in primitive myeloid cells during *Xenopus* embryogenesis

**Figure 1.**
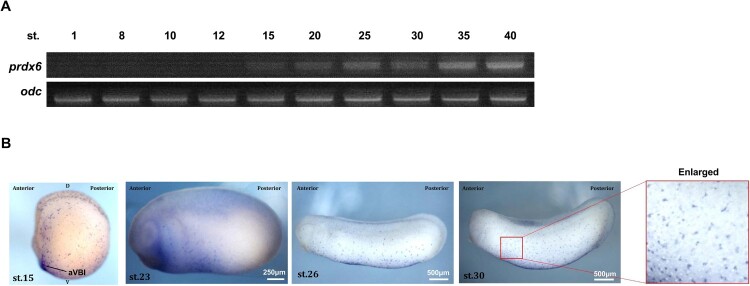
**Spatiotemporal expression pattern of *prdx6* during early *Xenopus* embryogenesis.** (A) Temporal expression of *prdx6* analyzed by RT-PCR. *prdx6* is a zygotic gene, with expression initiating at the neurula stage and increasing through the late tailbud stage. Ornithine decarboxylase (*odc*) was used as a loading control. (B) Spatial expression of *prdx6* detected by WISH. *prdx6* mRNA is specifically localized in primitive myeloid cells. Abbreviations: aVBI, anterior ventral blood island; D, dorsal; V, ventral. **Scale bars are shown in each panel.**

To investigate the spatiotemporal expression pattern of *prdx6*, we performed reverse transcription – polymerase chain reaction (RT–PCR) and whole-mount *in situ* hybridization (WISH). RT–PCR analysis revealed that *prdx6* expression commenced at the neurula stage and progressively increased through the late tailbud stages (Figure 1A). WISH analysis demonstrated that *prdx6* was specifically expressed in myeloid progenitors derived from the anterior ventral blood island (aVBI), starting at stage 15. As development progressed, the number of *prdx6*-expressing cells expanded throughout the embryo, consistent with the known distribution pattern of primitive myeloid cells (Figure 1B).

These findings suggest that Prdx6 plays a key role in the specification and differentiation of primitive myeloid cells during *Xenopus* embryogenesis.

### 3.2. *Prdx6* is required for maintaining the myeloid cell population

**Figure 2.**
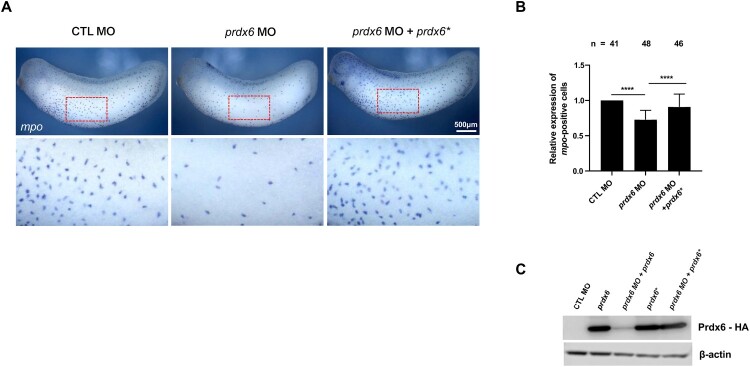
**Effect of *prdx6* knockdown on myeloid cell development.** (A) Embryos were injected with 60 ng of *prdx6* MOs at the one-cell stage and fixed at stage 28. WISH was performed using the myeloid-specific marker *mpo*. Compared with control MO-injected embryos, *prdx6* knockdown resulted in a significant reduction in the number of myeloid cells. *prdx6-*deficient phenotypes were rescued by co-injection with MO-resistant *prdx6* mRNA. (B) Quantification of *mpo*-positive myeloid cells in the epidermis. The number of *mpo*-positive cells was counted for each embryo, and the values were normalized to the average count in control embryos, which was set to 1. ‘n’ indicates the total number of embryos analyzed for each condition. (C) Western blot analysis of Prdx6 protein levels in embryos injected with control MO, *prdx6* MO, *prdx6* mRNA (*prdx6*), and MO-resistant mRNA (prdx6*), detected using an anti-HA antibody. β-actin was used as a loading control. **Scale bars are shown in each panel.**

To investigate the functional role of Prdx6 in early development, we employed a loss-of-function approach using a *prdx6*-targeted antisense morpholino oligonucleotide (MO). A total of 60 ng MO was injected into one-cell-stage embryos to inhibit *prdx6* translation. Knockdown efficiency was confirmed by co-injecting wild-type *prdx6* mRNA, which showed reduced Prdx6 protein expression (Figure 2C). Given the spatial expression of *prdx6*, we assessed its impact on myeloid cells using *mpo* as a molecular marker in WISH analysis. Knockdown of *prdx6* resulted in a significant reduction in *mpo*-positive myeloid cells compared to embryos injected with control MO (Figure 2A, B). To confirm that the observed phenotype was specifically due to *prdx6* depletion, we performed rescue experiments using an MO-resistant *prdx6* mRNA (prdx6*). Co-injection of prdx6* mRNA successfully restored the number of myeloid cells to levels comparable to those in the control (Figure 2A, B), thereby confirming that Prdx6 is required for maintaining the myeloid cell population.

### 3.3. Prdx6 is not essential for myeloid cell function

**Figure 3.**
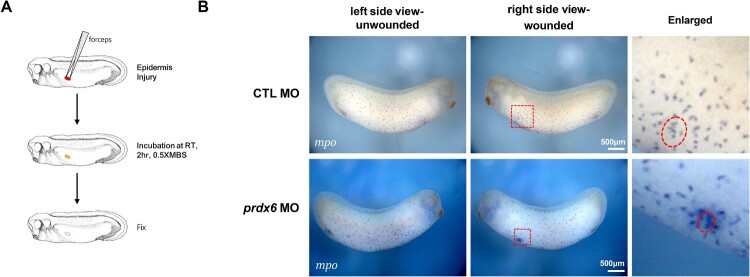
**Prdx6 is not required for the wound-healing response of primitive myeloid cells.** (A) Schematic diagram of the wound-healing assay. (B) WISH analysis of *mpo* expression in control MO-injected embryos and *prdx6* morphants. In both groups, myeloid cells were successfully recruited to wound sites. **Scale bars are shown in each panel**.

We examined whether Prdx6 is necessary for myeloid cell function, particularly in response to tissue damage. A wound-healing assay was performed by inducing epidermal wounds in stage 28 embryos and allowing time for cellular migration toward the injury site. Embryos were subsequently fixed and analyzed for *mpo* expression by WISH. In both control and *prdx6* morphants, myeloid cells migrated efficiently to the wound sites (Figure 3A, B). These results indicate that Prdx6 is not essential for the migratory behavior or wound response of primitive myeloid cells.

### 3.4. Prdx6 regulates ROS levels to control myeloid cell development

**Figure 4.**
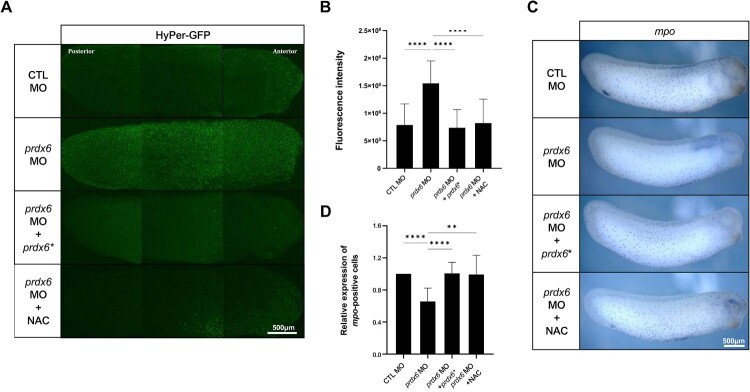
***Prdx6*-dependent ROS regulation controls primitive myeloid cell development.** (A) One-cell stage embryos were co-injected with HyPer mRNA (0.5 ng) and either *prdx6* MO or control MO. *Prdx6* knockdown resulted in increased fluorescence intensity, indicating elevated ROS levels. NAC (1 mM) was administered starting at stage 8. Both mRNA injection and NAC treatment restored ROS levels. (B) Quantification of HyPer-GFP fluorescence intensity using ImageJ. (C) Embryos were injected with *prdx6* MO or control MO, followed by NAC treatment (1 mM) from stage 8 onward. WISH was conducted using the myeloid-specific marker *mpo*. *prdx6* morphants exhibited a reduction in the number of myeloid cells compared to the controls. Co-injection of prdx6* mRNA or NAC treatment restored myeloid cell numbers. (D) Quantification of *mpo*-positive myeloid cells was performed as described in Figure 2B. **Scale bars are shown in each panel**.

ROS play an important regulatory role during embryonic development, and members of the Prdx family are central to redox homeostasis (Lee et al. 2021). Previous studies have shown that ROS regulation by Prdx proteins is essential for processes such as kidney development and ciliogenesis (Chae et al. 2017; Ji et al. 2019). To determine whether Prdx6 influences ROS levels, we used the HyPer-GFP system to monitor intracellular H₂O₂ levels in embryos (Lee et al. 2020). HyPer-GFP is a genetically encoded sensor that detects intracellular H₂O₂ through a redox-sensitive domain derived from OxyR. Upon oxidation, this domain undergoes a conformational change that alters GFP fluorescence intensity, thereby enabling the visualization of H₂O₂ levels in live embryos (Belousov et al. 2006). Based on this principle, we co-injected *prdx6* MO and HyPer-GFP mRNA into embryos and performed confocal imaging at the tailbud stage to quantify ROS levels. *Prdx6* morphants exhibited significantly increased ROS levels compared to control embryos, and this increase was reversed by co-injection of prdx6* mRNA (Figure 4A, B). Consistent with these findings, *prdx6* morphants showed a reduced number of *mpo*-positive myeloid cells (Figure 4C, D). This phenotype was rescued by co-injection of prdx6* mRNA or treatment with N-acetylcysteine (NAC), a known ROS scavenger, both of which also restored ROS levels (Figure 4). Collectively, these findings demonstrate that Prdx6 regulates the development of myeloid cells by maintaining intracellular ROS levels during embryogenesis.

### 3.5. Prdx6 promotes proliferation of primitive myeloid progenitors

**Figure 5.**
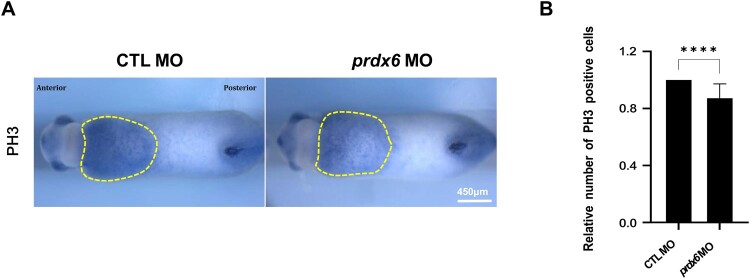
***Prdx6* deficiency impairs cell proliferation during myelopoiesis.** (A) Cell proliferation was assessed through PH3 staining in ventral views of control and *prdx6* morphants. (B) Quantitative analysis of PH3 staining intensity. The stained areas and grayscale values were measured using ImageJ. The relative proliferation index was calculated by dividing the stained area by the grayscale value and comparing *prdx6* MO to control MO. **Scale bars are shown in each panel**.

Myelopoiesis in *Xenopus* embryos begins at the neurula stage, during which progenitor cells originating from the aVBI differentiate and migrate by the early tailbud stage. Although *prdx6* morphants showed a wide distribution of myeloid cells (Figure 2A), the overall number of these cells was significantly reduced. This suggested a possible defect in proliferation rather than differentiation or migration. Previous studies have implicated that excessive ROS levels can inhibit cell cycle progression by triggering oxidative stress responses (Masgras et al. 2012; Wang et al. 2017). To further investigate this, we assessed cell proliferation in the aVBI using phospho-histone 3 (PH3) staining. *Prdx6*-depleted embryos exhibited a marked reduction in PH3-positive cells compared to controls (Figure 5). These findings indicate that Prdx6 promotes the proliferation of myeloid progenitors by maintaining redox homeostasis during early development.

## 4. Discussion

Our study reveals a previously uncharacterized role of Prdx6 in primitive myeloid cell development. Specifically, we show that Prdx6 regulates intracellular ROS levels, which are critical for the proliferation of myeloid progenitors. Although other members of the Prdx family have been linked to embryonic processes such as cilia formation and kidney morphogenesis (Chae et al., 2017; Ji et al., 2019), Prdx6’s contribution to hematopoiesis had not been demonstrated.

Multiple lines of evidence support this conclusion. First, *prdx6* is expressed zygotically, beginning at the neurula stage, coinciding with the onset of primitive myelopoiesis in the aVBI (Figure 1A). WISH analysis further confirmed that *prdx6* mRNA is spatially restricted to myeloid progenitors from stage 15 onward (Figure 1B). Second, *prdx6* knockdown led to a reduction in the myeloid cell numbers and an increase in ROS levels. Co-injection of prdx6* mRNA or treatment with NAC restored both parameters (Figures 2 and 4). These findings highlight the functional importance of Prdx6-mediated redox regulation during myeloid development.

Unlike other Prdx enzymes, Prdx6 possesses additional phospholipase A2 activity, suggesting a dual role in redox regulation and lipid metabolism (Fisher 2011; Fujita et al. 2024). Whether the observed developmental effects are attributable solely to peroxidase activity remains unclear. Future studies employing catalytic mutants will be valuable in elucidating the role of each enzymatic function.

Interestingly, *prdx6* morphants retained the ability to initiate myeloid cell migration and respond to injury (Figure 3), suggesting that Prdx6 does not affect cell motility. Notably, these embryos also exhibited an increased accumulation of myeloid cells at the wound site (Figure 3). Rather than indicating a direct enhancement of recruitment, this observation may reflect a secondary response to elevated ROS levels and inflammatory signaling resulting from *prdx6* knockdown. Such changes could amplify chemotactic signals in the tissue microenvironment, thereby promoting the localized clustering of the remaining myeloid cells (Kumin et al. 2007; Niethammer et al. 2009; Yang et al. 2022). In contrast, the overall reduction in the number of myeloid cells appears to be due to impaired proliferation, as evidenced by decreased PH3 staining (Figure 5). Although elevated ROS levels were correlated with this defect, further investigation is needed to establish a direct causal relationship.

Although myeloid cell depletion in *prdx6* morphants did not result in overt morphological abnormalities, previous studies have highlighted the crucial role of primitive macrophages in normal development and tissue remodeling (Savill and Fadok 2000; Smith and Mohun 2011). Beyond their developmental roles, myeloid cells also contribute significantly to tissue repair and regeneration (Aztekin et al. 2020), suggesting that Prdx6 may be involved in both embryogenesis and regenerative contexts.

In summary, our findings identify Prdx6 as a key antioxidant enzyme that promotes the proliferation of primitive myeloid cells through redox regulation. These insights advance our understanding of ROS-dependent developmental processes and may serve as a basis for investigating the role of Prdx6 in regenerative biology and disease.
